# *In Vitro* Activity of Eravacycline against Gram-Negative Bacilli Isolated in Clinical Laboratories Worldwide from 2013 to 2017

**DOI:** 10.1128/AAC.01699-19

**Published:** 2020-02-21

**Authors:** Ian Morrissey, Melanie Olesky, Stephen Hawser, Sibylle H. Lob, James A. Karlowsky, G. Ralph Corey, Matteo Bassetti, Corey Fyfe

**Affiliations:** a IHMA Europe Sàrl, Monthey, Switzerland; b Tetraphase Pharmaceuticals, Watertown, Massachusetts, USA; c International Health Management Associates, Inc., Schaumburg, Illinois, USA; d Department of Medical Microbiology and Infectious Diseases, Max Rady College of Medicine, University of Manitoba, Winnipeg, Manitoba, Canada; e Duke University Medical Center, Durham, North Carolina, USA; f Infectious Diseases Clinic, Department of Medicine, University of Udine, Udine, Italy

**Keywords:** eravacycline, multidrug resistant, MDR, Gram negative, *Enterobacteriaceae*, *Acinetobacter*

## Abstract

Eravacycline is a novel, fully synthetic fluorocycline antibiotic developed for the treatment of serious infections, including those caused by multidrug-resistant (MDR) pathogens. Here, we evaluated the *in vitro* activities of eravacycline and comparator antimicrobial agents against a global collection of frequently encountered clinical isolates of Gram-negative bacilli. The CLSI broth microdilution method was used to determine MIC data for isolates of *Enterobacterales* (*n* = 13,983), Acinetobacter baumannii (*n* = 2,097), Pseudomonas aeruginosa (*n* = 1,647), and Stenotrophomonas maltophilia (*n* = 1,210) isolated primarily from respiratory, intra-abdominal, and urinary specimens by clinical laboratories in 36 countries from 2013 to 2017. Susceptibilities were interpreted using both CLSI and EUCAST breakpoints. Multidrug-resistant (MDR) isolates were defined by resistance to agents from ≥3 different antimicrobial classes. The MIC_90_s ranged from 0.25 to 1 μg/ml for *Enterobacteriaceae* and were 1 μg/ml for A. baumannii and 2 μg/ml for S. maltophilia, Proteus mirabilis, and Serratia marcescens. Eravacycline’s potency was up to 4-fold greater than that of tigecycline against genera/species of *Enterobacterales*, A. baumannii, and S. maltophilia. The MIC_90_s for five of six individual genera/species of *Enterobacterales* and A. baumannii were within 2-fold of the MIC_90_s for their respective subsets of MDR isolates, while the MDR subpopulation of *Klebsiella* spp. demonstrated 4-fold higher MIC_90_s. Eravacycline demonstrated potent *in vitro* activity against the majority of clinical isolates of Gram-negative bacilli, including MDR isolates, collected over a 5-year period. This study further underscores the potential benefit of eravacycline in the treatment of infections caused by MDR Gram-negative pathogens.

## Abstract (precis)

Eravacycline is a novel, fully synthetic fluorocycline antibiotic developed for the treatment of serious infections, including those caused by multidrug-resistant (MDR) pathogens. Here, we evaluated the *in vitro* activities of eravacycline and comparator antimicrobial agents against a global collection of frequently encountered clinical isolates of Gram-negative bacilli. The CLSI broth microdilution method was used to determine MIC data for isolates of *Enterobacterales* (*n* = 13,983), Acinetobacter baumannii (*n* = 2,097), Pseudomonas aeruginosa (*n* = 1,647), and Stenotrophomonas maltophilia (*n* = 1,210) isolated primarily from respiratory, intra-abdominal, and urinary specimens by clinical laboratories in 36 countries from 2013 to 2017.

## INTRODUCTION

Gram-negative pathogens causing serious infections are becoming an increasing clinical concern (1–5). Important antimicrobial-resistant Gram-negative pathogens include extended-spectrum-β-lactamase (ESBL)-producing Escherichia coli and Klebsiella pneumoniae; carbapenemase-producing, fluoroquinolone-resistant, and multidrug-resistant (MDR) *Enterobacterales*; as well as carbapenem-resistant and MDR Pseudomonas aeruginosa, Acinetobacter baumannii, and Stenotrophomonas maltophilia (1–5). In fact, many of these pathogens have recently been highlighted among Gram-negative MDR pathogens classified as urgent/critical or serious/high public health threats by the Centers for Disease Control and Prevention (CDC) (1) and the World Health Organization (WHO) (6).

Eravacycline is a novel, fully synthetic fluorocycline antibiotic developed for the treatment of serious infections, including those caused by MDR pathogens (5, 7; https://clinicaltrials.gov/ct2/show/NCT01844856). Eravacycline is comprised of a tetracycline core with two novel modifications, a fluorine atom at the C-7 position and a pyrrolidinoacetamido group at the C-9 position, both of which are on the D ring (7, 9). These novel modifications confer enhanced *in vitro* activity compared to that of other tetracyclines against resistant Gram-negative and Gram-positive bacteria, and the pyrrolidinoacetamido group allows for increased ribosomal binding and steric hindrance to avoid ribosome protection-based tetracycline resistance.

Eravacycline inhibits bacterial protein synthesis (i.e., acyl-tRNA transfer) by binding to the 30S ribosomal subunit (10). Eravacycline demonstrates potent broad-spectrum activity against Gram-negative bacilli (except for P. aeruginosa and *Burkholderia* spp.) and Gram-positive cocci, including anaerobes, as well as atypical bacterial pathogens and Neisseria gonorrhoeae (5, 11–16), and does not exhibit a loss of antibacterial activity against isolates expressing tetracycline ribosomal protection genes or most tetracycline efflux resistance genes (10, 11, 14).

Eravacycline has successfully completed clinical trials for the treatment of complicated intra-abdominal infection (cIAI) (https://clinicaltrials.gov/ct2/show/NCT01844856) and has been approved for use by both the U.S. Food and Drug Administration (FDA) and the European Medicines Agency (EMA). Additionally, eravacycline has demonstrated *in vivo* efficacy as a treatment in murine models of systemic, thigh, and lung infection and pyelonephritis.

The objective of the current study was to determine the *in vitro* activity of eravacycline relative to that of other antimicrobial agents using a representative global collection of clinical isolates of Gram-negative bacilli.

## RESULTS AND DISCUSSION

A total of 17,781 Gram-negative aerobic isolates collected between 2013 and 2017 were included in this study. *Enterobacteriaceae* accounted for the majority of these isolates (*n* = 10,531). The MIC distributions for these isolates and the cumulative percentage of isolates inhibited by eravacycline are shown in Table 1. The MIC_90_ for isolates of A. baumannii was 1 μg/ml, and it was 0.5 μg/ml for the four genera/species of *Enterobacteriaceae* combined and 2 μg/ml for S. maltophilia. Among the six genera/species of *Enterobacterales* tested, MIC_90_ values were as follows: E. coli, 0.25 μg/ml; *Klebsiella* spp. and *Citrobacter* spp., 0.5 μg/ml; *Enterobacter* spp., 1 μg/ml; and Proteus mirabilis and Serratia marcescens, 2 μg/ml. For MDR populations of these organisms, the MIC_90_ values of eravacycline remained within 2- to 4-fold of the MIC_90_s indicated above. Given eravacycline’s limited activity against P. aeruginosa (Table 1), concordant with this pathogen’s established intrinsic resistance to tetracyclines, it was excluded from further analyses (17).

**TABLE 1:** Cumulative percentage of all clinical isolates of *Enterobacteriaceae*, individual genera/species of *Enterobacterales*, A. baumannii, P. aeruginosa, and S. maltophilia tested from 2013 to 2017 inhibited by eravacycline, by MIC

Organism	No. of isolates	Cumulative % of isolates inhibited by eravacycline at the following MIC (μg/ml)^*a*^:											
		≤0.015	0.03	0.06	0.12	0.25	0.5	1	2	4	8	16	>16
All isolates													
*Enterobacteriaceae*	10,531		<0.1	2.4%	30.0	73.2	92.6	96.9	98.9	99.8	>99.9	100	
*Klebsiella* spp.	4,965			0.7	23.2	73.0	90.6	95.8	98.6	99.7	>99.9	100	
*Enterobacter* spp.	1,820			<0.1	3.0	43.1	89.6	95.5	98.0	99.7	100		
*Citrobacter* spp.	1,776			1.0	37.4	81.9	94.6	98.3	99.8	100			
E. coli	1,970		0.2	9.9	65.4	94.0	98.8	99.7	100				
P. mirabilis	1,348				0.1	1.4	10.6	48.3	91.8	99.6	99.8	99.9	100
S. marcescens	948				0.1	0.6	10.3	67.1	95.6	99.3	100		
A. baumannii	2,097	1.0	7.3	18.4	28.9	42.3	70.5	92.5	98.7	99.6	99.9	100	
P. aeruginosa	1,647		0.1		0.2	0.3	1.0	2.9	6.7	37.2	78.2	96.6	100
S. maltophilia	1,210		0.1	0.4	2.8	13.5	41.7	77.9	93.6	98.9	99.9	100	
MDR isolates^*b*^													
*Enterobacteriaceae*	2,051		<0.1	1.5	18.3	55.0	80.5	91.3%	96.8	99.5	>99.9	100	
*Klebsiella* spp.	801			0.4	8.5	43.7	72.4	88.0	95.1	99.0	99.9	100	
*Enterobacter* spp.	448				1.6	32.6	73.4	86.4	94.4	99.3	100		
*Citrobacter* spp.	247			0.4	17.4	55.5	80.2	91.9	99.2	100			
E. coli	555		0.2	4.7	46.5	89.2	98.0	99.6	100				
P. mirabilis	328					0.6	7.9	37.5	82.9	98.8	99.1	99.7	100
S. marcescens	87						4.6	43.7	86.2	97.7	100		
A. baumannii	1,502		0.1	1.4	7.4	21.5	59.1	89.5	98.1	99.4	99.9	100	
P. aeruginosa	339						0.3	3.2	6.2	21.2	56.0	90.6	100

a The MIC_90_ is shaded gray.

b MDR was defined using CLSI breakpoints.

When isolates were allocated to their respective geographic regions, eravacycline MIC_50_s and MIC_90_s were within 1 doubling dilution for all *Enterobacteriaceae* combined, individual genera/species of *Enterobacterales*, A. baumannii, and S. maltophilia (see Table S1 in the supplemental material). Similarly, there were no significant differences (a >1-doubling-dilution increase or decrease in the MIC_50_s or MIC_90_s) observed in the *in vitro* activity of eravacycline for any genera/species of Gram-negative bacilli stratified by specimen source (Table S2) or stratified by study period (2013 to 2014, 2015, 2016, 2017) (Table S3). A detailed trend analysis could not be conducted, given that there were changes in participating laboratories and the panel of antimicrobial agents tested over the time period studied (2013 to 2017).

The *in vitro* activities of eravacycline and comparator agents against *Enterobacterales*, A. baumannii, and S. maltophilia isolates and their MDR counterparts are shown in Tables 2 and 3, respectively. The rates of susceptibility of *Enterobacteriaceae* spp. to eravacycline were high, with the susceptibility rates being 98.8% for E. coli, 90.6% for *Klebsiella* spp., 94.6% for *Citrobacter* spp., and 89.6% for *Enterobacter* spp. (Table 2) (18). For MDR organisms, the rates of susceptibility to eravacycline for E. coli remained high (97.5%) and ranged from 77 to 81.9% for the other *Enterobacteriaceae* (Table 3). Breakpoint interpretations were not available for eravacycline against the other organisms tested.

**TABLE 2:** *In vitro* activity of eravacycline and comparator agents against *Enterobacteriaceae*, individual genera/species of *Enterobacterales*, A. baumannii, and S. maltophilia, cumulative 2013 to 2017 data

Organism	Antimicrobial agent	No. of isolates	MIC (μg/ml)			% susceptible according to the following breakpoint:	
			50%	90%	Range	CLSI	EUCAST
*Enterobacteriaceae*	Eravacycline	10,531	0.25	0.5	0.03 to 16	92.6^*a*^	92.6
	Amikacin	7,534	1	4	≤0.25 to >64	99.1	98.3
	Aztreonam	10,531	0.25	>16	≤0.03 to >16	78.9	76.2
	Cefepime	10,531	0.12	8	≤0.008 to >16	87.9	85.5
	Cefotaxime	7,534	0.12	>64	≤0.015 to >64	76.2	76.2
	Ceftazidime	10,531	≤0.5	32	≤0.5 to >16	80.1	76.9
	Ceftriaxone	10,531	0.25	>4	≤0.5 to >4	76.4	76.4
	Colistin	10,531	0.5	1	≤0.12 to >4	NA^*c*^	99.4
	Ertapenem	7,534	0.015	0.25	≤0.002 to >2	94.2	94.2
	Gentamicin	10,531	0.5	2	≤0.25 to >8	91.2	90.7
	Imipenem	2,997	0.5	2	≤0.25 to >8	86.7	97.5
	Levofloxacin	10,531	0.12	>4	≤0.25 to >4	82.4	82.4
	Meropenem	7,534	0.03	0.06	≤0.004 to >4	97.9	98.2
	Minocycline	5,059	2	8	≤0.12 to >16	84.0	NA
	Piperacillin-tazobactam	10,531	2	64	≤0.5 to >64	84.3	79.9
	Tetracycline	10,531	2	64	≤0.25 to >8	80.3	NA
	Tigecycline	10,531	0.5	1	≤0.015 to 32	96.8^*a*^	70.6
	Trimethoprim-sulfamethoxazole	7,534	0.12	>4	≤0.06 to >4	81.5	81.5
*Klebsiella* spp.	Eravacycline	4,965	0.25	0.5	0.06 to 16	90.6	90.6
	Amikacin	3,472	1	2	≤0.25 to >64	98.5	97.8
	Aztreonam	4,965	≤0.5	>16	≤0.5 to >16	80.5	77.6
	Cefepime	4,965	≤0.25	8	≤0.25 to >16	88.7	87.4
	Cefotaxime	3,472	0.06	>64	≤0.015 to >64	78.9	78.9
	Ceftazidime	4,965	≤0.5	>16	≤0.5 to >16	82.4	79.4
	Ceftriaxone	4,965	≤0.5	>4	≤0.5 to >4	78.4	78.4
	Colistin	1,493	0.5	1	≤0.12 to >4	NA	98.2
	Ertapenem	3,472	0.015	0.25	≤0.002 to >2	94.7	94.7
	Gentamicin	4,965	≤0.25	1	≤0.25 to >8	92.6	92.3
	Imipenem	1,493	0.5	2	≤0.25 to >8	81.0	96.3
	Levofloxacin	4,965	≤0.25	2	≤0.25 to >4	85.7	85.7
	Meropenem	3,472	0.03	0.06	≤0.004 to >4	96.7	97.0
	Minocycline	2,324	2	8	≤0.12 to >16	83.3	NA
	Piperacillin-tazobactam	4,965	2	64	≤0.5 to >64	83.3	79.2
	Tetracycline	4,965	2	>8	≤0.25 to >8	84.1	NA
	Tigecycline	4,965	0.5	2	0.06 to 32	95.9	64.1
	Trimethoprim-sulfamethoxazole	3,472	0.12	>4	≤0.06 to >4	86.0	86.0
*Enterobacter* spp.	Eravacycline	1,820	0.5	1	0.06 to 8	89.6	89.6
	Amikacin	1,322	1	2	≤0.25 to >64	99.4	98.8
	Aztreonam	1,820	≤0.5	>16	≤0.5 to >16	68.6	66.0
	Cefepime	1,820	≤0.25	8	≤0.25 to >16	84.6	79.0
	Cefotaxime	1,322	0.5	>64	≤0.015 to >64	63.2	63.2
	Ceftazidime	1,820	≤0.5	>16	≤0.5 to >16	67.9	65.1
	Ceftriaxone	1,820	≤0.5	>4	≤0.5 to >4	63.7	63.7
	Colistin	498	0.5	1	≤0.12 to >4	NA	94.4
	Ertapenem	1,322	0.06	1	0.004 to >2	86.4	86.4
	Gentamicin	1,820	≤0.25	2	≤0.25 to >8	91.2	90.3
	Imipenem	498	0.5	1	≤0.25 to >8	91.6	97.2
	Levofloxacin	1,820	≤0.25	1	≤0.25 to >4	87.1	87.1
	Meropenem	1,322	0.06	0.12	0.008 to >4	98.3	98.8
	Minocycline	880	4	8	0.25 to >16	83.4	NA
	Piperacillin-tazobactam	1,820	4	>64	≤0.5 to >64	76.2	71.0
	Tetracycline	1,820	2	>8	0.5 to >8	83.8	NA
	Tigecycline	1,820	0.5	2	0.03 to 8	95.5	55.9
	Trimethoprim-sulfamethoxazole	1,322	0.12	>4	≤0.06 to >4	82.6	82.6
*Citrobacter* spp.	Eravacycline	1,776	0.25	0.5	0.06 to 4	94.6	94.6
	Amikacin	1,272	1	2	≤0.25 to >64	99.6	99.0
	Aztreonam	1,776	≤0.5	>16	≤0.5 to >16	82.3	80.2
	Cefepime	1,776	≤0.25	1	≤0.25 to >16	94.8	91.5
	Cefotaxime	1,272	0.12	64	≤0.015 to >64	79.5	79.5
	Ceftazidime	1,776	≤0.5	>16	≤0.5 to >16	82.0	79.1
	Ceftriaxone	1,776	≤0.5	>4	≤0.5 to >4	80.6	80.6
	Colistin	504	1	1	≤0.12 to >4	NA	99.0
	Ertapenem	1,272	0.008	0.25	≤0.002 to >2	96.1	96.1
	Gentamicin	1,776	0.5	1	≤0.25 to >8	95.7	95.4
	Imipenem	504	0.5	2	≤0.25 to >8	86.7	98.8
	Levofloxacin	1,776	≤0.25	0.5	≤0.25 to >4	90.0	90.0
	Meropenem	1,272	0.03	0.06	0.008 to >4	98.7	98.7
	Minocycline	881	2	8	≤0.12 to >16	87.2	NA
	Piperacillin-tazobactam	1,776	2	64	≤0.5 to >64	86.0	81.2
	Tetracycline	1,776	1	8	≤0.25 to >8	89.3	NA
	Tigecycline	1,776	0.5	1	≤0.015 to 4	97.8	80.5
	Trimethoprim-sulfamethoxazole	1,272	≤0.06	1	≤0.06 to >4	90.4	90.4
E. coli	Eravacycline	1,970	0.12	0.25	0.03 to 2	98.8	98.8
	Amikacin	1,468	2	4	≤0.25 to >64	99.7	98.5
	Aztreonam	1,970	≤0.5	>16	≤0.5 to >16	81.5	78.5
	Cefepime	1,970	≤0.25	>16	≤0.25 to >16	82.8	81.5
	Cefotaxime	1,468	0.06	>64	≤0.015 to >64	78.7	78.7
	Ceftazidime	1,970	≤0.5	16	≤0.5 to >16	84.2	79.8
	Ceftriaxone	1,970	≤0.5	>4	≤0.5 to >4	79.6	79.6
	Colistin	502	0.5	1	0.25 to 4	NA	99.4
	Ertapenem	1,468	0.008	0.06	≤0.002 to >2	98.7	98.7
	Gentamicin	1,970	0.5	>8	≤0.25 to >8	83.5	82.8
	Imipenem	502	≤0.25	0.5	≤0.25 to 2	99.0	100.0
	Levofloxacin	1,970	≤0.25	>4	≤0.25 to >4	62.8	62.8
	Meropenem	1,468	0.03	0.03	≤0.004 to 2	99.7	100.0
	Minocycline	974	1	16	≤0.12 to >16	83.5	NA
	Piperacillin-tazobactam	1,970	2	16	≤0.5 to >64	92.5	88.9
	Tetracycline	1,970	2	>8	≤0.25 to >8	59.4	NA
	Tigecycline	1,970	0.25	0.5	0.03 to 4	99.2	91.6
	Trimethoprim-sulfamethoxazole	1,468	0.12	>4	≤0.06 to >4	62.3	62.3
P. mirabilis	Eravacycline	1,348	2	2	0.12 to >16	10.6	10.6
	Amikacin	940	2	4	0.5 to >64	97.8	96.7
	Aztreonam	1,348	≤0.5	≤0.5	≤0.5 to >16	98.6	94.6
	Cefepime	1,348	≤0.25	1	≤0.25 to >16	94.2	92.2
	Cefotaxime	940	≤0.015	2	≤0.015 to >64	89.4	89.4
	Ceftazidime	1,348	≤0.5	≤0.5	≤0.5 to >16	94.4	91.8
	Ceftriaxone	1,348	≤0.5	2	≤0.5 to >4	89.9	89.9
	Colistin	408	>4	>4	4 to >4	NA	0.0
	Ertapenem	940	0.015	0.015	0.004 to >2	99.7	99.7
	Gentamicin	1,348	1	>8	≤0.25 to >8	84.9	79.1
	Imipenem	408	2	4	≤0.25 to >8	19.1	NA^*b*^
	Levofloxacin	1,348	≤0.25	>4	≤0.25 to >4	67.6	67.6
	Meropenem	940	0.06	0.12	0.008 to >4	99.7	99.8
	Minocycline	631	16	>16	1 to >16	2.2	NA
	Piperacillin-tazobactam	1,348	≤0.5	1	≤0.5 to >64	98.8	98.0
	Tetracycline	1,348	>8	>8	0.5 to >8	2.0	NA
	Tigecycline	1,348	4	8	0.25 to >32	26.7	1.3
	Trimethoprim-sulfamethoxazole	940	0.12	>4	≤0.06 to >4	64.2	64.2
S. marcescens	Eravacycline	948	1	2	0.12 to 8	10.3	10.3
	Amikacin	451	2	4	≤0.25 to >64	98.9	97.8
	Aztreonam	948	≤0.5	2	≤0.5 to >16	92.7	89.6
	Cefepime	948	≤0.25	0.5	≤0.25 to >16	95.5	94.4
	Cefotaxime	451	0.5	8	0.06 to >64	81.2	81.2
	Ceftazidime	948	≤0.5	1	≤0.5 to >16	95.9	93.0
	Ceftriaxone	948	≤0.5	4	≤0.5 to >4	87.1	87.1
	Colistin	497	>4	>4	1 to >4	NA	3.8
	Ertapenem	451	0.03	0.12	0.008 to >2	95.8	95.8
	Gentamicin	948	0.5	2	≤0.25 to >8	96.3	94.8
	Imipenem	497	1	2	≤0.25 to >8	88.1	98.6
	Levofloxacin	948	≤0.25	2	≤0.25 to >4	85.2	85.2
	Meropenem	451	0.06	0.12	0.008 to >4	97.1	97.1
	Minocycline	229	4	8	0.5 to >16	85.2	NA
	Piperacillin-tazobactam	948	2	16	≤0.5 to >64	91.6	87.7
	Tetracycline	948	>8	>8	2 to >8	3.2	NA
	Tigecycline	948	2	2	0.25 to 16	92.2	2.9
	Trimethoprim-sulfamethoxazole	451	0.25	1	≤0.06 to >4	93.4	93.4
A. baumannii	Eravacycline	2,097	0.5	1	≤0.015 to 16	NA	NA
	Amikacin	1,598	32	>64	0.12 to >64	48.9	46.5
	Ampicillin-sulbactam	1,598	32	>64	0.25 to >64	33.1	NA
	Aztreonam	2,097	>16	>16	≤0.03 to >16	NA	NA
	Cefepime	2,097	>16	>16	≤0.25 to >16	27.6	NA
	Ceftazidime	2,097	>16	>16	≤0.5 to >16	30.2	NA
	Ceftriaxone	2,097	>32	>32	≤0.03 to >32	12.7	NA
	Colistin	2,097	0.5	2	≤0.03 to >4	95.1	95.1
	Gentamicin	2,097	>8	>8	≤0.03 to >8	38.9	38.9
	Imipenem	499	>8	>8	≤0.25 to >8	37.3	37.3
	Levofloxacin	2,097	>4	>4	≤0.25 to >4	27.7	26.1
	Meropenem	1,598	32	>64	≤0.03 to >64	33.9	33.9
	Minocycline	1,598	2	16	≤0.03 to >64	67.3	NA
	Piperacillin-tazobactam	2,097	>64	>64	≤0.5 to >64	25.9	NA
	Tetracycline	2,097	>8	>8	≤0.25 to >8	26.8	NA
	Tigecycline	2,097	2	4	0.06 to >16	NA	NA
	Trimethoprim-sulfamethoxazole	1,598	16	>64	≤0.03 to >64	39.3	39.3
S. maltophilia	Eravacycline	1,210	1	2	0.03 to 16	NA	NA
	Amikacin	1,080	>64	>64	0.12 to >64	NA	NA
	Ampicillin-sulbactam	1,080	>64	>64	1 to >64	NA	NA
	Aztreonam	1,210	>16	>16	0.06 to >16	NA	NA
	Cefepime	1,210	>16	>16	0.06 to >16	NA	NA
	Ceftazidime	1,210	16	>16	≤0.5 to >16	39.2	NA
	Ceftriaxone	1,210	>32	>32	0.12 to >32	NA	NA
	Colistin	1,210	2	>4	≤0.12 to >4	NA	NA
	Gentamicin	1,210	>8	>8	≤0.25 to >8	NA	NA
	Imipenem	130	>8	>8	4 to >8	NA	NA
	Levofloxacin	1,210	1	>4	≤0.25 to >4	76.4	NA
	Meropenem	1,080	>64	>64	0.06 to >64	NA	NA
	Minocycline	1,080	0.5	2	0.06 to 64	99.0	NA
	Piperacillin-tazobactam	1,210	>64	>64	2 to >64	NA	NA
	Tetracycline	1,210	>8	>8	1 to >8	NA	NA
	Tigecycline	1,210	2	4	0.06 to >16	NA	NA
	Trimethoprim-sulfamethoxazole	1,080	0.5	4	≤0.03 to >64	84.3	90.3

a U.S. Food and Drug Administration (FDA) MIC interpretative breakpoints for *Enterobacteriaceae* for eravacycline (susceptible, ≤0.5 μg/ml) and tigecycline (susceptible, ≤2 μg/ml; intermediate, 4 μg/ml; resistant, ≥8 μg/ml) (20) were used in place of CLSI MIC breakpoints, as none currently exist. The 2019 EUCAST MIC interpretative breakpoints for tigecycline (susceptible, ≤0.5 μg/ml; resistant, ≥1 μg/ml) (19) are different from the FDA MIC breakpoints.

b The MIC panel range is not low enough for *Proteeae* for 2019 EUCAST breakpoints.

c NA, MIC breakpoint not available.

**TABLE 3:** *In vitro* activity of eravacycline and comparator agents against MDR *Enterobacteriaceae*, individual genera/species of *Enterobacterales*, and A. baumannii, cumulative 2013 to 2017 data

Organism	Antimicrobial agent	CLSI criteria					EUCAST criteria				
		No. of isolates	MIC (μg/ml)			% susceptible	No. of isolates	MIC (μg/ml)			% susceptible
			50%	90%	Range			50%	90%	Range	
All *Enterobacteriaceae*	Eravacycline	2,051	0.25	1	0.03 to 16	80.5^*a*^	2,186	0.25	1	0.06 to 16	82.0^*b*^
	Amikacin	1,656	2	8	≤0.25 to >64	96.0	1,614	2	8	≤0.25 to >64	92.5
	Aztreonam	2,051	>16	>16	≤0.5 to >16	18.4	2,186	>16	>16	≤0.03 to >16	6.2
	Cefepime	2,051	4	>16	≤0.25 to >16	42.1	2,186	4	>16	0.015 to >16	35.3
	Cefotaxime	1,656	>64	>64	≤0.015 to >64	17.5	1,614	>64	>64	≤0.015 to >64	8.1
	Ceftazidime	2,051	>16	>16	≤0.5 to >16	24.3	2,186	32	>16	≤0.03 to >16	9.0
	Ceftriaxone	2,051	>4	>4	≤0.5 to >4	14.7	2,186	>4	>4	≤0.015 to >4	6.0
	Colistin	2,051	0.5	1	≤0.12 to >4	NA^*c*^	2,186	0.5	1	≤0.12 to >4	98.7
	Ertapenem	1,656	0.12	>2	0.004 to >2	75.7	1,614	0.25	>2	0.004 to >2	73.9
	Gentamicin	2,051	1	>8	≤0.25 to >8	58.6	2,186	1	>8	≤0.12 to >8	62.1
	Imipenem	395	0.5	8	≤0.25 to >8	76.0	572	0.5	2	≤0.25 to >8	90.4
	Levofloxacin	2,051	4	>4	≤0.25 to >4	34.8	2,186	2	>8	0.008 to >4	43.4
	Meropenem	1,656	0.06	1	≤0.004 to >4	90.7	1,614	0.06	1	≤0.004 to >4	91.6
	Minocycline	1,112	4	>16	≤0.12 to >16	58.0	1,166	4	>16	≤0.12 to >16	NA
	Piperacillin-tazobactam	2,051	32	>64	≤0.5 to >64	44.2	2,186	64	>64	≤0.25 to >64	25.2
	Tetracycline	2,051	>8	>8	≤0.25 to >8	40.1	2,186	4	>8	≤0.25 to >8	NA
	Tigecycline	2,051	1	2	0.06 to 16	92.0^*a*^	2,186	0.5	2	0.06 to 16	51.7^*b*^
	Trimethoprim-sulfamethoxazole	1,656	>4	>4	≤0.06 to >4	37.6	1,614	>4	>4	≤0.06 to >4	45.9
*Klebsiella* spp.	Eravacycline	801	0.5	2	0.06 to 16	72.4	943	0.5	1	0.06 to 16	77.0
	Amikacin	626	2	16	≤0.25 to >64	92.0	667	2	16	≤0.25 to >64	88.6
	Aztreonam	801	>16	>16	≤0.5 to >16	8.6	943	>16	>16	≤0.03 to >16	3.4
	Cefepime	801	>16	>16	≤0.25 to >16	33.0	943	8	>16	0.015 to >16	37.7
	Cefotaxime	626	>64	>64	≤0.015 to >64	10.4	667	>64	>64	≤0.015 to >64	7.2
	Ceftazidime	801	>16	>16	≤0.5 to >16	19.5	943	>16	>128	≤0.03 to >128	10.1
	Ceftriaxone	801	>4	>4	≤0.5 to >4	6.9	943	>4	>32	0.03 to >32	3.7
	Colistin	175	1	1	0.25 to >4	NA	276	0.5	1	0.25 to >4	94.2
	Ertapenem	626	0.25	>2	0.008 to >2	72.2	667	0.25	>2	0.008 to >2	73.3
	Gentamicin	801	1	>8	≤0.25 to >8	57.4	943	1	>16	≤0.12 to >16	62.1
	Imipenem	175	1	>8	≤0.25 to >8	68.0	276	1	8	≤0.25 to >8	86.2
	Levofloxacin	801	4	>4	≤0.25 to >4	32.6	943	1	>8	0.03 to >8	43.9
	Meropenem	626	0.06	>4	0.015 to >4	82.1	667	0.06	>4	0.015 to >4	84.4
	Minocycline	438	4	>16	0.25 to >16	50.7	484	4	>16	0.25 to >16	NA
	Piperacillin-tazobactam	801	64	>64	≤0.5 to >64	31.0	943	64	>128	0.5 to >128	17.1
	Tetracycline	801	8	>8	≤0.25 to >8	46.2	943	4	>64	≤0.25 to >64	NA
	Tigecycline	801	1	4	0.12 to 16	89.5	943	1	2	0.12 to 16	38.7
	Trimethoprim-sulfamethoxazole	626	>4	>4	≤0.06 to >4	35.9	667	>4	>4	≤0.06 to >4	42.9
*Enterobacter* spp.	Eravacycline	448	0.5	2	0.12 to 8	73.4	529	0.5	2	0.12 to 8	78.5
	Amikacin	364	1	4	≤0.25 to >64	97.8	381	1	4	≤0.25 to >64	96.1
	Aztreonam	448	>16	>16	≤0.5 to >16	10.0	529	>16	>16	0.06 to >16	1.9
	Cefepime	448	4	>16	≤0.25 to >16	43.3	529	4	>16	0.06 to >16	31.2
	Cefotaxime	364	>64	>64	0.06 to >64	6.6	381	>64	>64	0.12 to >64	0.5
	Ceftazidime	448	>16	>16	≤0.5 to >16	9.8	529	64	>128	0.25 to >128	1.0
	Ceftriaxone	448	>4	>4	≤0.5 to >4	6.0	529	>4	>32	0.5 to >32	0.6
	Colistin	84	0.5	1	≤0.12 to 2	NA	148	0.5	1	≤0.12 to >4	95.3
	Ertapenem	364	0.5	>2	0.015 to >2	56.0	381	0.5	>2	0.015 to >2	54.1
	Gentamicin	448	0.5	>8	≤0.25 to >8	65.2	529	0.5	>16	≤0.12 to >16	69.0
	Imipenem	84	1	8	≤0.25 to >8	75.0	148	0.5	2	≤0.25 to >8	91.9
	Levofloxacin	448	0.5	>4	≤0.25 to >4	54.5	529	0.25	8	0.008 to >8	63.5
	Meropenem	364	0.12	0.5	0.015 to >4	94.2	381	0.12	0.5	0.015 to >4	95.8
	Minocycline	227	4	>16	1 to >16	62.1	272	4	>16	1 to >16	NA
	Piperacillin-tazobactam	448	64	>64	≤0.5 to >64	31.3	529	64	>128	≤0.5 to >128	13.0
	Tetracycline	448	4	>8	1 to >8	50.7	529	4	>64	0.5 to >64	NA
	Tigecycline	448	1	4	0.12 to 8	87.5	529	1	4	0.12 to 8	42.3
	Trimethoprim-sulfamethoxazole	364	4	>4	≤0.06 to >4	49.7	381	0.5	>4	≤0.06 to >4	59.6
*Citrobacter* spp.	Eravacycline	247	0.25	1	0.06 to 4	80.2	282	0.25	1	0.06 to 4	81.9
	Amikacin	210	1	8	≤0.25 to >64	98.1	222	1	4	≤0.25 to >64	95.1
	Aztreonam	247	>16	>16	≤0.5 to >16	18.6	282	>16	>16	0.06 to >16	4.3
	Cefepime	247	2	>16	≤0.25 to >16	67.6	282	1	>16	0.06 to >16	51.4
	Cefotaxime	210	64	>64	0.06 to >64	13.3	222	64	>64	0.12 to >64	3.6
	Ceftazidime	247	>16	>16	≤0.5 to >16	15.4	282	128	>128	0.5 to >128	1.8
	Ceftriaxone	247	>4	>4	≤0.5 to >4	12.6	282	>4	>32	0.12 to >32	3.9
	Colistin	37	1	2	0.25 to >4	NA	60	1	1	0.25 to >4	96.7
	Ertapenem	210	0.25	2	0.004 to >2	76.7	222	0.25	2	0.008 to >2	77.5
	Gentamicin	247	0.5	>8	≤0.25 to >8	72.5	282	0.5	>16	≤0.12 to >16	78.0
	Imipenem	37	1	8	≤0.25 to >8	62.2	60	1	2	≤0.25 to >8	91.7
	Levofloxacin	247	0.5	>4	≤0.25 to >4	53.4	282	0.25	>8	0.015 to >8	60.6
	Meropenem	210	0.06	1	0.015 to >4	91.9	222	0.06	1	0.015 to >4	92.8
	Minocycline	139	8	>16	≤0.12 to >16	48.9	162	4	>16	≤0.12 to >16	NA
	Piperacillin-tazobactam	247	>64	>64	≤0.5 to >64	27.1	282	64	>128	≤0.25 to >128	9.9
	Tetracycline	247	4	>8	≤0.25 to >8	53.9	282	2	>64	≤0.25 to >64	NA
	Tigecycline	247	0.5	2	0.12 to 4	92.7	282	0.5	2	0.12 to 4	58.9
	Trimethoprim-sulfamethoxazole	210	0.25	>4	≤0.06 to >4	57.6	222	0.12	>4	≤0.06 to >4	67.1
E. coli	Eravacycline	555	0.25	0.5	0.03 to 2	98.0	432	0.25	0.5	0.06 to 2	97.5
	Amikacin	456	2	8	≤0.25 to >64	99.1	344	2	8	0.5 to >64	94.5
	Aztreonam	555	16	>16	≤0.5 to >16	39.1	432	>16	>16	≤0.03 to >16	18.8
	Cefepime	555	8	>16	≤0.25 to >16	43.1	432	16	>16	0.015 to >16	24.5
	Cefotaxime	456	>64	>64	≤0.015 to >64	37.9	344	>64	>64	≤0.015 to >64	21.2
	Ceftazidime	555	8	>16	≤0.5 to >16	46.9	432	16	64	0.06 to >128	21.1
	Ceftriaxone	555	>4	>4	≤0.5 to >4	34.1	432	>4	>32	≤0.015 to >32	18.8
	Colistin	99	0.5	1	0.25 to 2	NA	88	0.5	1	0.25 to 4	96.6
	Ertapenem	456	0.015	0.25	0.004 to >2	95.8	344	0.03	0.25	0.004 to >2	94.5
	Gentamicin	555	8	>8	≤0.25 to >8	48.8	432	>8	>16	0.25 to >16	43.1
	Imipenem	99	≤0.25	1	≤0.25 to 2	96.0	88	≤0.25	1	≤0.25 to 2	100.0
	Levofloxacin	555	>4	>4	≤0.25 to >4	13.9	432	8	>8	0.03 to >8	6.3
	Meropenem	456	0.03	0.06	≤0.004 to 2	99.1	344	0.03	0.06	≤0.004 to 2	100.0
	Minocycline	308	2	16	0.25 to >16	69.5	248	2	16	0.25 to >16	NA
	Piperacillin-tazobactam	555	4	64	≤0.5 to >64	81.3	432	4	>64	≤0.25 to >128	67.8
	Tetracycline	555	>8	>8	0.5 to >8	16.8	432	64	>64	0.5 to >64	NA
	Tigecycline	555	0.25	1	0.06 to 4	98.9	432	0.25	1	0.06 to 4	87.0
	Trimethoprim-sulfamethoxazole	456	>4	>4	≤0.06 to >4	20.8	344	>4	>4	≤0.06 to >4	23.0
P. mirabilis	Eravacycline	328	2	4	0.25 to >16	7.9	191	2	4	0.25 to >16	6.3
	Amikacin	241	2	16	0.5 to >64	91.3	128	2	>64	0.5 to >64	79.7
	Aztreonam	328	≤0.5	4	≤0.5 to >16	94.8	191	≤0.5	4	≤0.03 to >16	67.5
	Cefepime	328	≤0.25	16	≤0.25 to >16	77.7	191	1	>16	0.03 to >16	53.4
	Cefotaxime	241	0.03	>64	≤0.015 to >64	61.0	128	32	>64	≤0.015 to >64	35.9
	Ceftazidime	328	≤0.5	>16	≤0.5 to >16	78.1	191	1	32	≤0.03 to >128	53.9
	Ceftriaxone	328	≤0.5	>4	≤0.5 to >4	62.5	191	4	16	≤0.015 to >32	43.5
	Colistin	87	>4	>4	>4 to >4	NA	63	>4	>4	>4 to >4	0.0
	Ertapenem	241	0.015	0.03	0.004 to >2	98.8	128	0.015	0.06	0.004 to >2	97.7
	Gentamicin	328	8	>8	≤0.25 to >8	48.8	191	>8	>16	0.25 to >16	15.7
	Imipenem	87	4	8	≤0.25 to >8	8.1	63	4	8	≤0.25 to >8	NA^*b*^
	Levofloxacin	328	>4	>4	≤0.25 to >4	11.0	191	>4	>8	0.03 to >8	5.8
	Meropenem	241	0.06	0.12	0.015 to >4	99.2	128	0.06	0.25	0.015 to >4	98.4
	Minocycline	167	16	>16	8 to >16	0.0	93	>16	>16	8 to >16	NA
	Piperacillin-tazobactam	328	≤0.5	8	≤0.5 to >64	95.4	191	1	8	≤0.25 to >128	90.6
	Tetracycline	328	>8	>8	1 to >8	0.6	191	32	>64	1 to >64	NA
	Tigecycline	328	4	8	0.25 to >32	21.0	191	4	8	0.25 to >32	1.1
	Trimethoprim-sulfamethoxazole	241	>4	>4	≤0.06 to >4	7.1	128	>4	>4	≤0.06 to >4	7.0
S. marcescens	Eravacycline	87	2	4	0.5 to 8	4.6	85	2	4	0.5 to 8	5.9
	Amikacin	54	2	16	1 to >64	90.7	38	4	32	1 to >64	79.0
	Aztreonam	87	16	>16	≤0.5 to >16	36.8	85	16	>16	≤0.5 to >16	7.1
	Cefepime	87	2	>16	≤0.25 to >16	54.0	85	2	>16	≤0.25 to >16	41.2
	Cefotaxime	54	32	>64	0.25 to >64	25.9	38	64	>64	0.5 to >64	2.6
	Ceftazidime	87	4	>16	≤0.5 to >16	58.6	85	4	32	0.25 to >128	34.1
	Ceftriaxone	87	>4	>4	≤0.5 to >4	20.7	85	>4	>32	1 to >32	2.4
	Colistin	33	>4	>4	1 to >4	NA	47	>4	>4	1 to >4	6.4
	Ertapenem	54	0.12	>2	0.015 to >2	72.2	38	0.25	>2	0.03 to >2	63.2
	Gentamicin	87	2	>8	≤0.25 to >8	60.9	85	2	>16	≤0.12 to >16	52.9
	Imipenem	33	1	4	≤0.25 to >8	75.8	47	1	2	≤0.25 to >8	91.5
	Levofloxacin	87	2	>4	≤0.25 to >4	26.4	85	2	8	0.06 to >8	30.6
	Meropenem	54	0.12	>4	0.008 to >4	83.3	38	0.12	>4	0.03 to >4	79.0
	Minocycline	17	4	16	2 to >16	52.9	18	4	16	2 to >16	NA
	Piperacillin-tazobactam	87	16	>64	1 to >64	52.9	85	32	>128	2 to >128	25.9
	Tetracycline	87	>8	>8	4 to >8	3.5	85	>8	32	4 to >64	NA
	Tigecycline	87	2	4	0.5 to 8	77.0	85	2	4	0.5 to 4	1.2
	Trimethoprim-sulfamethoxazole	54	2	>4	0.12 to >4	50.0	38	4	>4	0.12 to >4	44.7
A. baumannii	Eravacycline	1,502	0.5	2	0.03 to 16	NA	1,271	0.5	2	0.06 to 16	NA
	Amikacin	1,130	>64	>64	0.12 to >64	27.8	1,007	>64	>64	0.12 to >64	17.8
	Ampicillin-sulbactam	1,130	64	>64	1 to >64	7.3	1,007	64	>64	2 to >64	NA
	Aztreonam	1,502	>16	>16	0.25 to >16	NA	1,271	>64	>64	8 to >64	NA
	Cefepime	1,502	>16	>16	≤0.25 to >16	3.4	1,271	64	>64	2 to >64	NA
	Ceftazidime	1,502	>16	>16	≤0.5 to >16	4.4	1,271	>64	>64	2 to >64	NA
	Ceftriaxone	1,502	>32	>32	1 to >32	0.9	1,271	>64	>64	8 to >64	NA
	Colistin	1,502	0.5	2	≤0.03 to >4	NA	1,271	0.5	2	0.12 to >32	92.5
	Gentamicin	1,502	>8	>8	≤0.03 to >8	17.6	1,271	>64	>64	0.06 to >64	8.9
	Imipenem	372	>8	>8	0.5 to >8	15.9	264	>8	>8	8 to >8	0.0
	Levofloxacin	1,502	>4	>4	≤0.25 to >4	2.4	1,271	16	>64	0.06 to >64	0.1
	Meropenem	1,130	64	>64	0.06 to >64	7.9	1,007	64	>64	0.5 to >64	4.7
	Minocycline	1,130	4	16	≤0.03 to >64	54.0	1,007	4	16	≤0.03 to >64	NA
	Piperacillin-tazobactam	1,502	>64	>64	≤0.5 to >64	1.8	1,271	>128	>128	2 to >128	NA
	Tetracycline	1,502	>8	>8	0.5 to >8	4.5	1,271	>64	>64	2 to >64	NA
	Tigecycline	1,502	4	8	0.25 to >16	NA	1,271	4	8	0.25 to >16	NA
	Trimethoprim-sulfamethoxazole	1,130	64	>64	≤0.03 to >64	15.8	1,007	64	>64	0.06 to >64	11.1

a U.S. Food and Drug Administration (FDA) MIC interpretative breakpoints for *Enterobacteriaceae* for eravacycline (susceptible, ≤0.5 μg/ml) and tigecycline (susceptible, ≤2 μg/ml; intermediate, 4 μg/ml; resistant, ≥8 μg/ml) (20) were used in place of CLSI MIC breakpoints, as none currently exist. The 2019 EUCAST MIC interpretative breakpoints for tigecycline (susceptible, ≤0.5 μg/ml; resistant, ≥1 μg/ml) (19) are different from the FDA MIC breakpoints.

b The MIC panel range is not low enough for *Proteeae* for 2019 EUCAST breakpoints.

c NA, MIC breakpoint not available.

With regard to the comparator agents tested, the rate of susceptibility among the *Enterobacteriaceae* by the use of CLSI criteria was the highest for amikacin (99.1%), tigecycline (96.8%), the carbapenems meropenem (97.9%) and ertapenem (94.2%), and gentamicin (91.2%) (Table 2). Similar susceptibility was observed using EUCAST guidelines, with exceptions existing, such as for minocycline and tetracycline, for which no EUCAST breakpoints are given, and for colistin (99.4% susceptible by the use of EUCAST breakpoints), for which CLSI breakpoints are not given. Also, by use of the EUCAST criteria, tigecycline susceptibility was reduced to 70.6%, whereas eravacycline susceptibility remained at 92.6%. Other *Enterobacterales*, P. mirabilis, and S. marcescens were distinct, with reduced susceptibility to the tetracycline class, especially when susceptibility was evaluated using EUCAST breakpoints (Table 2). Among the *Enterobacteriaceae*, 19.5% (*n* = 2,051) were defined as being MDR isolates using CLSI breakpoints and 20.8% (*n* = 2,186) were defined as being MDR isolates using EUCAST breakpoints. The rates of susceptibility to all other comparators except amikacin, colistin (EUCAST breakpoints only), ertapenem, gentamicin (EUCAST breakpoints only), imipenem, meropenem, and tigecycline (CLSI breakpoints only) were less than 60% for the MDR isolate population. These susceptibilities are similar to those seen in other global surveillance studies (19, 20).

In addition to the *Enterobacterales*, a large collection of Gram-negative nonfermenters was tested, including A. baumannii (*n* = 2,097) and S. maltophilia (*n* = 1,210). For A. baumannii, high rates of resistance to the majority of the comparators were seen, with the levels of susceptibility to colistin (95.1%) and minocycline (67.3%) being the highest. Among the few agents with a breakpoint for S. maltophilia, the rate of susceptibility to minocycline was the highest at 99.0%. Approximately 24% and 15.7% of the isolates were resistant to levofloxacin and trimethoprim-sulfamethoxazole, respectively, similar to the findings of a surveillance study looking at isolates from 1998 to 2008 in Taiwan (21).

In this surveillance study, eravacycline demonstrated improved potency, based on MIC_90_ values, over tigecycline, showing a 4-fold greater potency than tigecycline against populations of A. baumannii, *Klebsiella* spp., and P. mirabilis and a 2-fold greater potency against E. coli, *Enterobacter* spp., *Citrobacter* spp., and S. maltophilia. Eravacycline and tigecycline showed equivalent potency against S. marcescens. Previous *in vitro* studies comparing eravacycline and tigecycline have reported similar 2- to 4-fold improvements in the MIC_90_ values of eravacycline over those of tigecycline (7, 12, 13, 16, 22–26). The tigecycline MIC_90_s for *Enterobacterales* spp., A. baumannii, and S. maltophilia determined in this study were equivalent to the MIC_90_s of tigecycline determined in other global surveillance studies (27, 28).

In addition to greater potency, eravacycline presents additional potential advantages over tigecycline: higher concentrations in serum and tissue (especially in the lung) and improved tolerability (7, 29–31). Previous studies have demonstrated that the *in vitro* activity of eravacycline is not affected by the presence of many of the resistance mechanisms likely present in the current surveillance population. These include studies which looked at the *in vitro* activity of eravacycline against ESBL-producing isolates of E. coli and K. pneumoniae; fluoroquinolone-susceptible and fluoroquinolone-resistant isolates of E. coli (including those of the MDR sequence type 131 genotype); polymyxin-resistant (*mcr-1*-positive) *Enterobacteriaceae*; carbapenem-resistant *Enterobacteriaceae*, including KPC-, SME-, VIM-, IMP-, NDM-, and OXA-48-producing isolates; and MDR *Enterobacteriaceae* and A. baumannii (12, 13, 16, 22–24, 26).

Eravacycline is currently approved for use by both EMA and FDA for the treatment of complicated intra-abdominal infections. As previously described, eravacycline maintains an overall potency advantage over tigecycline across approved organisms. The adoption of restrictive breakpoint criteria for tigecycline and eravacycline in the EUCAST 2019 guidelines, in contrast to the broad, albeit decade-old, tigecycline breakpoints granted by the FDA, makes comparisons of susceptibility between the two agents difficult. While the FDA breakpoints for both agents are granted for *Enterobacteriaceae*, the clinical efficacy of eravacycline against E. coli, Enterobacter cloacae, Citrobacter freundii, and species of *Klebsiella* is noted. Current eravacycline breakpoints from EUCAST were published concurrently with a reduction in both the tigecycline breakpoint and the approved organism list. The disconnect between approved interpretative criteria and organism coverage for tigecycline and other agents across these regulatory agencies highlights the need for greater efforts toward the harmonization of breakpoints.

Treatment of complicated intra-abdominal infection (cIAI) involves a combination of surgery and empirical antimicrobial therapy. Antimicrobial therapy must be sufficient to encompass a wide range of pathogens, including Gram-positive and Gram-negative aerobic and anaerobic bacteria (32–34). In cIAI patients, the most commonly encountered Gram-negative pathogens include E. coli, *Klebsiella* spp., and *Enterobacter* spp. (35). A. baumannii is less common in cIAI infections but has been noted to be an increasing cause of postoperative infections in hospital settings (36–38).

Eravacycline demonstrated potent *in vitro* activity against the clinically important Gram-negative organisms associated with cIAIs in humans. For all organisms, including A. baumannii and S. maltophilia, the MIC_90_s of eravacycline were 2- to 4-fold lower than those of tigecycline. This global surveillance investigation highlights the broad-spectrum potency of eravacycline against Gram-negative bacteria, including MDR strains, and further underscores its potential benefit for the treatment of cIAIs and other polymicrobial infections caused by resistant pathogens.

## MATERIALS AND METHODS

### Bacterial isolates.

From 2013 through 2017, clinical isolates of *Enterobacterales* (*n* = 13,983), A. baumannii (*n* = 2,097), P. aeruginosa (*n* = 1,647), and S. maltophilia (*n* = 1,210) were collected by laboratories in 36 countries (in the Asia-Pacific region, Australia, Hong Kong, Japan, South Korea, Malaysia, New Zealand, Philippines, Singapore, Taiwan, Thailand, and Vietnam; in Europe, Austria, Belgium, Croatia, Czech Republic, Denmark, France, Germany, Greece, Hungary, Ireland, Italy, Latvia, Lithuania, Netherlands, Poland, Portugal, Romania, Russia, Serbia, Spain, Sweden, Switzerland, Turkey, and the United Kingdom; and in North America [the United States]). In the Asia-Pacific region, isolates were collected only in 2015 through 2017, and some other countries also did not participate in all years.

Table S1 in the supplemental material summarizes the numbers of isolates of *Enterobacteriaceae*, A. baumannii, and S. maltophilia collected by geographic region. For the *Enterobacteriaceae* and A. baumannii, approximately 40 to 45% of the isolates came from North America and Europe and 15% came from the Asia-Pacific region. For S. maltophilia, approximately 50% of the isolates came from Europe, 30% came from North America, and 20% came from the Asia-Pacific region. In total, there were 6,559, 5,667, 5,305, and 1,406 isolates, respectively, from respiratory, intra-abdominal, urinary, and other specimen sources.

The identity of each isolate was confirmed using matrix-assisted laser desorption ionization–time of flight (MALDI-TOF) mass spectrometry (Bruker Biotyper; Bruker Daltonics, Bremen, Germany). Isolates were limited to one per patient, determined by the participating laboratory algorithms to be clinically significant, and collected irrespective of their antimicrobial susceptibility profile and independently of patient gender or age. The study was not designed to directly compare the prevalence of antimicrobial-resistant pathogens across specific geographic locations but, rather, was designed to evaluate the *in vitro* activities of eravacycline and comparator antimicrobial agents against a global collection of frequently encountered clinical isolates of Gram-negative bacilli collected from 2013 to 2017.

### Antimicrobial susceptibility testing.

The *in vitro* susceptibilities of isolates were determined using the CLSI-defined broth microdilution method in 96-well broth microdilution panels (17, 39). The antimicrobial agents used in panel production were acquired as laboratory-grade powders from their respective manufacturers or from a commercial source. The list of antimicrobial agents tested in each of the four study periods varied slightly, in that some agents, in addition to those tested in the 2013 to 2014 period, were included in the 2015 to 2017 testing periods. Ten antimicrobial agents (aztreonam, cefepime, ceftazidime, ceftriaxone, eravacycline, gentamicin, levofloxacin, piperacillin-tazobactam, tetracycline, and tigecycline) were tested against all isolates in each study period from 2013 to 2017. Of note, imipenem was tested in 2013 and 2014, while ertapenem and meropenem were tested in 2015 to 2017; colistin was tested against *Enterobacteriaceae* only in 2013 and 2014; amoxicillin-clavulanate was tested only in 2015; ampicillin-sulbactam was tested only in 2015 and 2016; and cefotaxime, minocycline, and trimethoprim-sulfamethoxazole were tested only in 2015 to 2017. The eravacycline MICs for Gram-negative bacilli were read following the current CLSI standard for dilution method testing; MIC endpoints were read following panel incubation at 35°C in ambient air for 16 to 20 h (*Enterobacteriaceae*, P. aeruginosa) or 20 to 24 h (A. baumannii, S. maltophilia) (17). Quality control testing for eravacycline and other antimicrobial agents was performed on each day of testing as specified by the CLSI (39) using the CLSI-defined control strains E. coli ATCC 25922 and P. aeruginosa ATCC 27853.

MICs were interpreted using 2019 CLSI MIC breakpoints (17) and 2019 EUCAST MIC breakpoints (18), with the following exceptions: FDA MIC interpretative breakpoints for eravacycline and tigecycline (29) were used in place of CLSI MIC breakpoints, which are not currently published for these agents. Additionally, tigecycline breakpoints were lowered with the 2019 EUCAST MIC guidelines, while simultaneously, the label was restricted to E. coli and Citrobacter koseri from the *Enterobacteriaceae*; in order to perform a more accurate and complete comparison between eravacycline and tigecycline susceptibilities, the current breakpoints for both eravacycline and tigecycline were applied to susceptibility percentages for all *Enterobacteriaceae* species.

An MDR phenotype was defined as resistance to one or more agents from three or more antimicrobial agent classes recommended for testing for a specific pathogen or pathogen family (*Enterobacteriaceae*) and possessing MIC interpretative breakpoints published by CLSI (17) or EUCAST (18). Eravacycline and tigecycline were excluded from the list of agents used for the MDR determination. Because the list of antimicrobial agents tested in each study year varied slightly, the identified MDR populations also varied slightly in each study year. Identification of MDR isolates of S. maltophilia was excluded from the analysis because of the very limited number of antimicrobial agents with either CLSI (17) or EUCAST (18) MIC breakpoints for that species. Resistance to levofloxacin and trimethoprim-sulfamethoxazole was captured for this organism, as these represent the current treatment of choice and a proposed alternative, respectively (40).

## Supplementary Material

Supplemental file 1
