# Supplementary material for: In Vitro Activity of Eravacycline against Gram-Negative Bacilli Isolated in Clinical Laboratories Worldwide from 2013 to 2017
Source: Antimicrob Agents Chemother. 2020 Feb 21;64(3):e01699-19. doi: 10.1128/AAC.01699-19 (PMC7038303; doi:10.1128/AAC.01699-19)
Supplement: Supplemental file 1 [file AAC.01699-19-s0001.pdf]

SUPPLEMENTAL TABLE 1 *In vitro* activity of eravacycline and selected comparator agents against Gram-negative bacilli stratified by geographic region, cumulative 2013-2017 data

		µg/ml													
			Eravacycline		Tigecycline		Tetracycline		Minocycline			Meropenem			
Isolates	Geographic region	*n*[a]	MIC_{50}	MIC_{90}	MIC_{50}	MIC_{90}	MIC_{50}	MIC_{90}	*n*[b]	MIC_{50}	MIC_{90}	*n*[b]	MIC_{50}	MIC_{90}
All *Enterobacteriaceae*	Asia/Pacific[c]	1629	0.25	0.5	0.5	1	2	>8	1004	2	16	1629	0.03	0.12
	Europe	3933	0.25	0.5	0.5	1	2	>8	1942	2	8	2887	0.03	0.12
	North America	4969	0.25	0.5	0.5	1	2	>8	2113	2	8	3018	0.03	0.06
Klebsiella spp.	Asia/Pacific[c]	796	0.25	0.5	0.5	1	1	>8	471	2	16	796	0.03	0.06
	Europe	1694	0.25	0.5	0.5	2	2	>8	812	2	16	1,247	0.03	0.12
	North America	2475	0.25	0.5	0.5	2	2	>8	1,041	2	8	1,429	0.03	0.06
Enterobacter spp.	Asia/Pacific[c]	203	0.25	1	0.5	2	2	>8	146	4	8	203	0.06	0.25
	Europe	770	0.5	1	0.5	2	2	>8	415	4	8	622	0.06	0.12
	North America	847	0.5	0.5	0.5	1	2	8	319	2	8	497	0.03	0.12
Citrobacter spp.	Asia/Pacific[c]	317	0.12	0.5	0.5	1	1	>8	218	2	8	317	0.03	0.06
	Europe	788	0.25	0.5	0.5	1	1	4	326	1	4	490	0.03	0.06
	North America	671	0.25	0.5	0.5	1	1	8	337	2	8	465	0.03	0.06
E. coli	Asia/Pacific[c]	313	0.12	0.25	0.25	0.5	4	>8	169	2	8	313	0.03	0.03
	Europe	681	0.12	0.25	0.25	0.5	2	>8	389	1	16	528	0.03	0.03
	North America	976	0.12	0.25	0.25	1	2	>8	416	1	16	627	0.03	0.03
P. mirabilis	Asia/Pacific[c]	95	1	2	4	8	>8	>8	70	16	>16	95	0.06	0.12
	Europe	705	2	2	4	8	>8	>8	383	16	>16	555	0.06	0.12
	North America	548	1	2	4	8	>8	>8	178	16	>16	290	0.06	0.12
S. marcescens	Asia/Pacific[c]	45	1	1	2	2	>8	>8	19	4	4	45	0.06	0.06
	Europe	349	1	2	2	2	>8	>8	87	4	8	199	0.06	0.12
	North America	554	1	2	2	2	>8	>8	123	4	8	207	0.06	0.12
A. baumannii	Asia/Pacific[c]	287	0.5	1	2	4	>8	>8	287	1	16	287	64	>64
	Europe	849	0.5	1	2	8	>8	>8	699	4	16	699	64	>64
	North America	961	0.5	1	2	4	>8	>8	612	1	8	612	4	>64
S. maltophilia	Asia/Pacific[c]	244	1	2	2	4	>8	>8	244	0.5	2	244	>64	>64

	Europe	623	1	2	2	4	>8	>8	524	0.5	2	524	>64	>64
	North America	343	1	2	2	4	>8	>8	312	0.5	2	312	>64	>64
MDR isolates[d]														
All *Enterobacteriaceae*	Asia/Pacific[c]	458	0.25	1	0.5	2	>8	>8	297	4	> 16	458	0.06	0.5
	Europe	823	0.5	1	1	2	>8	>8	430	4	> 16	643	0.06	2
	North America	770	0.25	1	0.5	2	>8	>8	385	4	> 16	555	0.03	1
Klebsiella spp.	Asia/Pacific[c]	192	0.25	1	1	4	>8	>8	122	4	>16	192	0.06	>4
	Europe	350	0.5	2	1	2	8	>8	185	8	>16	263	0.06	>4
	North America	259	0.5	2	1	4	4	>8	131	4	>16	171	0.06	>4
Enterobacter spp.	Asia/Pacific[c]	69	0.5	2	1	4	4	>8	47	4	>16	69	0.12	0.5
	Europe	214	0.5	2	1	4	>8	>8	116	4	>16	181	0.12	0.5
	North America	165	0.5	2	1	4	4	>8	64	4	>16	114	0.06	0.25
Citrobacter spp.	Asia/Pacific[c]	69	0.25	1	0.5	2	4	>8	46	4	>16	69	0.06	1
	Europe	87	0.5	1	0.5	2	2	>8	40	4	>16	69	0.06	0.25
	North America	91	0.25	2	1	2	8	>8	53	8	>16	72	0.06	4
E. coli	Asia/Pacific[c]	128	0.12	0.25	0.25	0.5	>8	>8	82	2	16	128	0.03	0.06
	Europe	172	0.12	0.5	0.25	1	>8	>8	89	2	>16	130	0.03	0.06
	North America	255	0.25	0.5	0.5	1	>8	>8	137	2	16	198	0.03	0.06
P. mirabilis	Asia/Pacific[c]	26	1	4	4	16	>8	>8	17	16	>16	26	0.06	0.12
	Europe	190	2	4	4	8	>8	>8	115	16	>16	153	0.06	0.12
	North America	112	2	4	4	8	>8	>8	35	>16	>16	62	0.06	0.12
S. marcescens	Asia/Pacific[c]	5	--	--	--	--	--	--	1	--	--	5	--	--
	Europe	45	2	4	2	4	>8	>8	8	--	--	28	0.12	0.25
	North America	37	2	4	2	4	>8	>8	8	--	--	21	0.06	>4
A. baumannii	Asia/Pacific[c]	202	0.5	1	2	4	>8	>8	202	4	16	202	64	>64
	Europe	695	0.5	1	4	8	>8	>8	572	8	16	572	64	>64
	North America	605	0.5	2	2	8	>8	>8	356	2	16	356	64	>64

[a] The number of isolates tested against eravacycline, tetracycline, tigecycline, and piperacillin-tazobactam. All four of these agents were included in the study from 2013 to 2016.
[b] No. of organisms tested against minocycline (not tested in 2013-2014 and only tested against non-fermentative Gram-negative bacilli in 2015) and meropenem (not tested in 2013-2014).
[c] Isolates from the Asia/Pacific geographic region were included in the study in 2015 and 2016 only.
[d] MDR isolates defined using CLSI breakpoints.

SUPPLEMENTAL TABLE 2 *In vitro* activity of eravacycline and selected comparator agents against Gram-negative bacilli stratified by specimen source, cumulative 2013-2017 data

			μg/ml											
			Eravacycline		Tigecycline		Tetracycline		Minocycline			Meropenem		
Isolates	Specimen source	*n*[a]	MIC_{50}	MIC_{90}	MIC_{50}	MIC_{90}	MIC_{50}	MIC_{90}	*n*[b]	MIC_{50}	MIC_{90}	*n*[b]	MIC_{50}	MIC_{90}
All *Enterobacteriaceae*	Intra-abdominal	3740	0.25	0.5	0.5	2	2	>8	1614	2	8	2391	0.03	0.12
	Respiratory	2875	0.25	0.5	0.5	2	2	>8	1728	2	8	2583	0.03	0.06
	Urinary	3198	0.25	0.5	0.5	1	2	>8	1716	2	8	2559	0.03	0.06
Klebsiella spp.	Intra-abdominal	1610	0.25	1	0.5	2	2	>8	659	2	8	999	0.03	0.06
	Respiratory	1594	0.25	0.5	0.5	2	1	>8	943	2	8	1,373	0.03	0.06
	Urinary	1392	0.25	0.5	0.5	2	1	>8	722	2	8	1,100	0.03	0.06
Enterobacter spp.	Intra-abdominal	679	0.5	1	0.5	2	2	>8	320	2	8	493	0.06	0.12
	Respiratory	562	0.5	0.5	0.5	2	2	>8	350	4	8	518	0.03	0.12
	Urinary	465	0.5	1	0.5	2	2	>8	209	4	>16	310	0.06	0.12
Citrobacter spp.	Intra-abdominal	581	0.25	0.5	0.5	1	1	>8	284	2	16	401	0.03	0.06
	Respiratory	392	0.25	0.5	0.5	1	1	4	246	1	8	365	0.03	0.06
	Urinary	697	0.25	0.5	0.5	1	1	8	351	1	8	506	0.03	0.06
E. coli	Intra-abdominal	870	0.12	0.25	0.25	0.5	2	>8	351	1	8	498	0.03	0.03
	Respiratory	327	0.12	0.25	0.25	0.5	4	>8	189	2	16	327	0.03	0.03
	Urinary	644	0.12	0.25	0.25	1	2	>8	434	1	16	643	0.03	0.03
P. mirabilis	Intra-abdominal	411	2	2	4	8	>8	>8	177	16	>16	273	0.06	0.12
	Respiratory	290	2	4	4	8	>8	>8	195	16	>16	267	0.06	0.12
	Urinary	546	1	2	4	8	>8	>8	259	16	>16	400	0.06	0.12
S. marcescens	Intra-abdominal	151	1	2	2	2	>8	>8	30	4	4	59	0.06	0.12
	Respiratory	517	1	2	2	2	>8	>8	167	4	8	327	0.06	0.12
	Urinary	178	1	2	2	4	>8	>8	32	4	16	65	0.06	0.12
A. baumannii	Intra-abdominal	265	0.5	1	2	8	>8	>8	170	4	16	170	64	>64
	Respiratory	1,280	0.5	1	2	8	>8	>8	1,120	2	16	1,120	64	>64
	Urinary	373	0.25	1	2	4	>8	>8	305	0.5	16	305	2	>64
S. maltophilia	Intra-abdominal	180	1	2	1	4	>8	>8	134	0.5	2	134	>64	>64

	Respiratory	896	1	2	2	4	>8	>8	865	0.5	2	865	>64	>64
	Urinary	111	0.5	1	1	4	>8	>8	78	0.5	2	78	>64	>64
MDR isolates [c]														
All *Enterobacteriaceae*	Intra-abdominal	730	0.25	2	1	2	>8	>8	334	4	> 16	515	0.06	2
	Respiratory	573	0.25	1	0.5	2	>8	>8	373	4	> 16	545	0.06	4
	Urinary	667	0.25	1	0.5	2	>8	>8	405	4	> 16	596	0.06	0.25
Klebsiella spp.	Intra-abdominal	248	0.5	2	1	4	>8	>8	113	8	>16	158	0.06	>4
	Respiratory	257	0.25	1	1	2	4	>8	173	4	>16	242	0.06	>4
	Urinary	256	0.25	2	1	4	8	>8	152	4	>16	226	0.06	>4
Enterobacter spp.	Intra-abdominal	169	0.5	2	1	4	4	>8	84	4	>16	139	0.12	0.5
	Respiratory	142	0.5	2	1	4	8	>8	85	4	>16	131	0.06	0.5
	Urinary	124	0.5	2	1	4	4	>8	58	8	>16	94	0.06	0.25
Citrobacter spp.	Intra-abdominal	107	0.25	2	0.5	2	4	>8	57	4	>16	91	0.06	1
	Respiratory	48	0.25	1	0.5	2	2	>8	35	8	>16	46	0.06	2
	Urinary	84	0.25	1	0.5	2	8	>8	47	8	>16	73	0.06	0.25
E. coli	Intra-abdominal	206	0.25	0.5	0.25	0.5	>8	>8	80	2	>16	127	0.03	0.06
	Respiratory	126	0.12	0.25	0.25	1	>8	>8	80	2	16	126	0.03	0.12
	Urinary	203	0.25	0.5	0.25	1	>8	>8	148	2	16	203	0.03	0.06
P. mirabilis	Intra-abdominal	85	2	4	4	8	>8	>8	45	16	>16	59	0.06	0.25
	Respiratory	75	2	4	4	8	>8	>8	53	16	>16	73	0.06	0.5
	Urinary	150	2	4	4	8	>8	>8	69	>16	>16	109	0.06	0.12
S. marcescens	Intra-abdominal	13	2	4	2	4	>8	>8	2	--	--	7	--	--
	Respiratory	45	2	4	2	4	>8	>8	11	4	16	35	0.12	>4
	Urinary	23	1	2	2	4	>8	>8	4	--	--	12	0.12	>4
A. baumannii	Intra-abdominal	213	0.5	1	2	8	>8	>8	137	8	16	137	64	>64
	Respiratory	951	0.5	2	4	8	>8	>8	828	4	16	828	64	>64
	Urinary	205	0.5	1	4	8	>8	>8	162	4	16	162	64	>64

[a] No. of organisms tested against eravacycline, tetracycline, tigecycline, and piperacillin-tazobactam, which were all included in the study in 2013-2016.

[b] No. of organisms tested against minocycline (not tested in 2013-2014 and only tested against non-fermentative Gram-negative bacilli in 2015) and meropenem (not tested in 2013-2014).

[c] MDR isolates defined using CLSI breakpoints.

--, MIC_{50} and MIC_{90} not shown when n<10.

SUPPLEMENTAL TABLE 3 *In vitro* activity of eravacycline and selected comparator agents against Gram-negative bacilli stratified by study period

			μg/ml									
			Eravacycline		Tigecycline		Tetracycline		Minocycline		Meropenem	
Isolates	Study period	*n*	MIC_{50}	MIC_{90}	MIC_{50}	MIC_{90}	MIC_{50}	MIC_{90}	MIC_{50}	MIC_{90}	MIC_{50}	MIC_{90}
All *Enterobacteriaceae*	2013-2014	2,997	0.25	0.5	0.5	1	2	>8	--	--	--	--
	2015	2,475	0.25	0.5	0.5	1	2	>8	--	--	0.03	0.06
	2016	2,299	0.25	0.5	0.5	2	1	>8	2	16	0.03	0.06
	2017	2,643	0.25	0.5	0.5	2	1	>8	2	8	0.03	0.12
Klebsiella spp.	2013-2014	1,493	0.25	1	0.5	1	2	8	--	--	--	--
	2015	1,148	0.25	0.5	0.5	2	2	>8	--	--	0.03	0.06
	2016	1,057	0.25	0.5	0.5	2	1	>8	2	8	0.03	0.06
	2017	1,267	0.25	0.5	0.5	2	1	>8	2	8	0.03	0.12
Enterobacter spp.	2013-2014	498	0.5	1	0.5	1	2	>8	--	--	--	--
	2015	442	0.5	0.5	1	2	2	>8	--	--	0.06	0.12
	2016	476	0.25	0.5	0.5	2	2	>8	2	8	0.06	0.12
	2017[a]	404	0.25	0.5	0.5	2	2	>8	4	8	0.06	0.12
Citrobacter spp.	2013-2014	504	0.25	0.5	0.5	0.5	1	4	--	--	--	--
	2015	391	0.25	0.5	0.5	2	2	>8	--	--	0.03	0.06
	2016	380	0.12	0.5	0.25	1	1	8	2	8	0.03	0.06
	2017	501	0.25	0.5	0.5	1	1	4	1	8	0.03	0.06
E. coli	2013-2014	502	0.12	0.25	0.25	0.5	2	>8	--	--	--	--
	2015	494	0.12	0.5	0.5	0.5	2	>8	--	--	0.03	0.03
	2016	503	0.12	0.25	0.25	0.5	2	>8	1	16	0.03	0.03
	2017	471	0.12	0.25	0.25	1	2	>8	1	8	0.015	0.03
P. mirabilis	2013-2014	408	1	2	2	4	>8	>8	--	--	--	--
	2015	309	2	4	4	8	>8	>8	--	--	0.06	0.12
	2016	267	1	2	4	8	>8	>8	16	>16	0.06	0.12
	2017	364	1	2	4	8	>8	>8	16	>16	0.06	0.12
S. marcescens	2013-2014	497	1	2	2	2	>8	>8	--	--	--	--
	2015	222	1	2	2	4	>8	>8	--	--	0.06	0.12
	2016	183	1	2	1	2	>8	>8	4	8	0.06	0.12

	2017	46	1	2	2	4	>8	>8	4	8	0.06	0.06
A. baumannii	2013-2014	499	0.5	1	1	4	>8	>8	--	--	--	--
	2015	598	0.5	2	2	4	>8	>8	2	16	32	>64
	2016	503	0.5	1	4	8	>8	>8	2	16	64	>64
	2017	497	0.25	1	2	8	>8	>8	1	16	32	>64
S. maltophilia	2013-2014	130	0.5	1	0.5	2	>8	>8	--	--	--	--
	2015	560	1	2	2	4	>8	>8	0.5	2	64	>64
	2016	469	1	2	2	4	>8	>8	1	2	>64	>64
	2017	51	0.5	1	1	2	>8	>8	0.25	1	>64	>64
MDR isolates [b]												
All *Enterobacteriaceae*	2013-2014	395	0.5	2	1	2	>8	>8	--	--	--	--
	2015	544	0.5	1	1	2	>8	>8	--	--	0.06	0.25
	2016	513	0.25	1	0.5	2	>8	>8	4	>16	0.06	1
	2017	587	0.25	1	0.5	2	>8	>8	4	>16	0.06	> 4
Klebsiella spp.	2013-2014	175	0.5	2	1	2	8	>8	--	--	--	--
	2015	188	0.5	2	1	4	4	>8	--	--	0.06	>4
	2016	196	0.25	2	1	4	>8	>8	4	>16	0.06	4
	2017	242	0.25	1	1	2	8	>8	4	>16	0.06	>4
Enterobacter spp.	2013-2014	84	0.5	4	1	4	8	>8	--	--	--	--
	2015	137	0.5	2	1	4	4	>8	--	--	0.06	0.25
	2016	107	0.5	2	1	4	4	>8	4	>16	0.12	0.5
	2017[a]	120	0.5	2	1	4	4	>8	4	>16	0.12	2
Citrobacter spp.	2013-2014	37	0.25	1	0.5	2	2	>8	--	--	--	--
	2015	71	0.5	2	1	4	4	>8	--	--	0.06	0.25
	2016	66	0.25	1	0.5	2	4	>8	4	>16	0.06	>4
	2017	73	0.25	1	1	2	8	>8	8	>16	0.06	1
E. coli	2013-2014	99	0.25	0.5	0.25	0.5	>8	>8	--	--	--	--
	2015	148	0.25	0.5	0.5	1	>8	>8	--	--	0.03	0.12
	2016	156	0.12	0.25	0.25	1	>8	>8	2	>16	0.03	0.06
	2017	152	0.25	0.25	0.25	0.5	>8	>8	2	16	0.03	0.06
P. mirabilis	2013-2014	87	2	4	4	8	>8	>8	--	--	--	--
	2015	74	2	4	8	8	>8	>8	--	--	0.06	0.12

	2016	75	1	4	4	8	>8	>8	>16	>16	0.12	0.25
	2017	92	2	4	4	8	>8	>8	16	>16	0.06	0.12
S. marcescens	2013-2014	33	2	4	2	4	>8	>8	--	--	--	--
	2015	37	2	2	2	4	>8	>8	--	--	0.06	>4
	2016	16	1	4	2	4	>8	>8	4	16	0.12	>4
	2017	1	--	--	--	--	--	--	--	--	--	--
A. baumannii	2013-2014	372	0.5	1	2	4	>8	>8	--	--	--	--
	2015	429	1	2	4	8	>8	>8	4	16	64	>64
	2016	370	0.5	1	4	8	>8	>8	4	16	64	>64
	2017	331	0.5	1	4	8	>8	>8	4	16	64	>64

[a] 2017 *Enterobacter* spp. *E. cloacae* only.
[b] MDR isolates defined using CLSI breakpoints.
--, not tested or n<10.
